# Nomenclatural revision of *Cryptantha* (Boraginaceae s. str.) names linked to South American taxa

**DOI:** 10.3897/phytokeys.181.69740

**Published:** 2021-08-30

**Authors:** Pablo Moroni, Agustina Martínez, Michael G. Simpson

**Affiliations:** 1 Instituto de Botánica Darwinion (ANCEFN-CONICET), Labardén 200, CC 22, B1642HYD, San Isidro, Buenos Aires, Argentina Instituto de Botánica Darwinion Buenos Aires Argentina; 2 Department of Biology, San Diego State University, San Diego, California 92182, USA San Diego State University San Diego United States of America

**Keywords:** Boraginaceae, Johnston, nomenclature, Philippi, typification

## Abstract

During the preparation of the treatment of the genus *Cryptantha* Lehmann ex G. Don for South America, numerous names were identified as needing typification to stabilize their nomenclature. As a result, lectotypes are designated for 11 names and second-step lectotypes for 20 names. Furthermore, supporting information about the type material of the basionyms of four *Cryptantha* names already typified by Johnston (*Eritrichiumtalquinum* Phil., *Eritrichiumdimorphum* Phil., *Eritrichiumcarrizalense* Phil., and *Eritrichiumsubamplexicaule* Phil.) is provided.

## Introduction

Boraginaceae (sensu Weigend et al. 2014; Luebert et al. 2016; Hasenstab-Lehman 2017) are widely distributed, mainly in tropical, subtropical and temperate regions, with a high number of poorly studied taxa in the Americas. These lands are among the most diverse ecoregions in the world and, in line with this, their plant diversity has been a challenge, especially for taxonomically complex and diverse genera such as *Cryptantha* Lehmann ex G. Don. This genus and close relatives, commonly known in English as “popcorn flowers”, are notable for their simplified morphology, complex taxonomy and nomenclature, and partially resolved phylogeny (Mabry and Simpson 2018). Members of *Cryptantha* are strigose and/or hispid, annual or perennial herbs, with simple to highly branched, generally ascending to erect (rarely decumbent) stems and simple, basal to cauline, generally linear, lanceolate, or oblanceolate leaves (Johnston 1925; Payson 1927; Higgins 1971; Simpson and Hasenstab-Lehman 2009; Kelley et al. 2012).

As in the case of several other South American genera (e.g., O’Leary et al. 2007, 2010; Moroni et al. 2016; O’Leary et al. 2016), *Cryptantha* has a distinctive amphitropical distribution restricted to the non-tropical regions of western North America and western South America (Guilliams et al. 2017; Simpson et al. 2017). In these last lands, approximately 46 *Cryptantha* species distributed through Argentina, Bolivia, Chile and Peru (Johnston 1927; Zuloaga et al. 2008; Amsinckiinae Working Group 2021) are generally accepted (Amsinckiinae Working Group 2021), with a remainder of ca. 35 *Cryptantha* names considered synonymous with other *Cryptantha* taxa. At this point it is worth noting that, of the South American taxa of *Cryptantha*, the following have more recently undergone taxonomic changes: 1) *Cryptanthaalbida* (Kunth) I.M.Johnst. (which also occurs in North America), *C.diplotricha* (Phil.) Reiche, and *C.parviflora* (Phil.) Reiche have been transferred to the genus *Johnstonella* (Hasenstab-Lehman and Simpson 2012; Simpson et al. 2019); 2) *Cryptanthacircumscissa* (Hook. & Arn.) I.M. Johnst. has been transferred to the genus *Greeneocharis* (Hasenstab-Lehman and Simpson 2012); and 3) *Cryptanthaspegazzinii* I.M. Johnst. is a synonym of *Amsinckiacalycina* (Moris) Chater (Chater 1971).

Almost all of the currently accepted South American taxa of *Cryptantha* were treated by Johnston (1927), who provided a treatment based on his studies of Boraginaceae (see also Johnston 1925, 1935). After Johnston’s (1927) contribution, restricted taxonomic revisions of *Cryptantha* have been provided in regional floristic works for Argentina [covering Buenos Aires (Dawson 1965), Entre Ríos (Pérez Moreau 1979), Jujuy (Pérez-Moreau and Cabrera 1983), Patagonia (Correa 1999), and San Juan (Pérez-Moreau and Crespo 2018)]. Besides, floristic treatments and catalogues of *Cryptantha* have also been published for Argentina (Zuloaga and Morrone 1999), Bolivia (Miller et al. 2014), Chile (Rodríguez et al. 2018), and Peru (Macbride 1960; Brako and Zarucchi 1993), and for the Americas (Ulloa Ulloa et al. 2017) and the Southern Cone of the Americas (Zuloaga et al. 2008; Zuloaga et al. 2019).

Despite the taxonomic effort of the works mentioned above, the treatments proposed lack clarifications on nomenclatural subjects concerning the taxa studied, all of which constitute a non-trivial prerequisite to any comprehensive revisionary work. In light of this, the nomenclature of “one of the largest and most perplexing genera of the Boraginaceae” (Johnston 1927) still remains at the preliminary stage. Consequently, during the revision of *Cryptantha* for South America (Moroni, in prep.), a relatively large number of names were identified as needing typification or nomenclatural clarifications. Thus, the objective of this article is to make progress in producing a well-founded nomenclatural treatment for the genus in South America.

## Materials and methods

In order to resolve typifications, the protologues of the treated taxa were studied and key literature (e.g., Johnston 1927; Pérez-Moreau 1976) was consulted to identify possible prior typifications. Type specimens and original material from the herbaria CORD, E, F, G, GH, HAL, K, MA, P, S, SGO, and US (herbarium acronyms after Thiers 2021+) were analyzed from images on the JSTOR Global Plants database (ITHAKA 2021) or by personal communication with herbarium curators. To proceed with the typifications, the rules of the ICN (Turland et al. 2018) and suggestions proposed by McNeill (2014) were followed. In selecting lectotypes, whenever choosing between syntypes (Art. 9.6 of the ICN), the one that shows the best quality of preservation of the important diagnostic features of the taxon was selected to preserve the current application of the names involved.

Concerning the names described by the Prussian botanist R. A. Philippi, who greatly contributed to the taxonomic knowledge of *Cryptantha* in South America (Philippi 1857, 1860, 1864, 1873, 1891, 1895), a clarification of the material he used to describe numerous taxa might help to overcome some future difficulties. A relevant type collection of Philippi’s names is well-known to be currently lodged at SGO (Stafleu and Cowan 1983). However, duplicates annotated and thus presumably studied by him can be traced in several other herbaria such as CORD, GH, and HAL, or previously lodged at B and then destroyed by the fire caused by the Allied bombing in 1943 (Hiepko 1987; R. Vogt, curator at B, pers. comm.). In this context, it is not at all clear in which instances there is a holotype for his names, unless the author made clear in the protologue that only a single specimen of the gathering existed. Thereby, if Philippi did not specify a single specimen and syntypes are (or were) available, it is possible to designate a lectotype (Art. 40.2 of the ICN, Turland et al. 2018).

## Typifications

Lectotypes are here selected for 11 names, whereas 20 names already typified by Johnston (1927) or Pérez-Moreau (1976) were found to require second-step lectotypifications (Art. 9.17 of the ICN; Turland et al. 2018) because more than one specimen exists at the herbarium cited by them. It is worth noting that several names typified by Johnston are in need of second-step lectotypification given that the material held at SGO was unmounted and part of it was in storage by the time he visited the herbarium (Muñoz Pizarro 1960; Taylor and Muñoz-Schick 1994). On the other hand, supporting information related to the names *Eritrichiumtalquinum* Phil., *Eritrichiumdimorphum* Phil., *Eritrichiumcarrizalense* Phil., and *Eritrichiumsubamplexicaule* Phil. is provided.

Type designations are organized into a single treatment arranged by accepted species, with a full accounting of homotypic synonyms, followed by the heterotypic synonyms (and their homotypic synonyms) in need of typification, and a discussion of the typification/s involved. Concerning numerous specimens currently found at GH, they mainly consist of fragments removed by Johnston from sheets at P and SGO; *Amsinckiapatagonica* Speg. and *Cryptanthaargentea* I.M. Johnst. constitute the only exception in which the material lodged at GH consist of whole specimens.

### 1. Cryptantha alfalfalis

Taxon classificationPlantaeBoraginalesBoraginaceae

(Phil.) I.M. Johnst., Contr. Gray Herb. 78: 61. 1927.

696D3D25-B0A7-5935-8A5A-64BBAED6703E

≡ Eritrichiumalfalfalis Phil., Anales Univ. Chile 90: 525. 1895. Type: Chile. [Región Metropolitana de Santiago:] Río Colorado, Jan. 1888, *R.A. Philippi s.n.* (first-step lectotype, designated by Johnston 1927, pg. 61: SGO; second-step lectotype, designated here: SGO [SGO000004033 digital image!]; isolectotypes: SGO [SGO000004034 digital image!], GH [GH00096302 digital image!]).
= Eritrichiumrigidum Phil., Anales Univ. Chile 90: 529. 1895. Cryptantharigida (Phil.) Reiche, Fl. Chile 5: 224. 1907. Type: Chile. [Región Metropolitana de Santiago:] Río Colorado, Jan. 1888, *R.A. Philippi s.n.* (first-step lectotype, designated by Johnston 1927, pg. 61: SGO; second-step lectotype, designated here: SGO [SGO000004131 digital image!]; isolectotypes: GH [GH00096574 digital image!], SGO [SGO000004132 digital image!]).

#### Notes.

Rudolph A. Philippi’s (1895) description of *Eritrichiumalfalfalis* was based on a collection he made in the valley of the Colorado River in Chile. Johnston (1927) discussed this name and its original material, stating that the “type” was lodged at SGO, although two sheets annotated in Philippi’s hand as “Eritrichiumalfalfalis” are actually lodged there. The material found at SGO is in agreement with the locality and the diagnosis cited in the protologue. Thus, according to the Art. 9.17 (Turland et al. 2018), the choice of Johnston (1927) is here interpreted as a first-step lectotypification. From among the material available for typification purposes, the sheet SGO000004033 is here selected as a second-step lectotype of the name.

The protologue of *Eritrichiumrigidum* (Philippi, 1895) includes a direct reference to a collection made by R. A. Philippi in the valley of the Colorado River in Chile. Johnston (1927) referred to the original material of this name, stating that the “type” was lodged at SGO. However, two sheets annotated by Philippi as “Eritrichiumrigidum” were found there in agreement with the protologue. Thus, Johnston’s statement is here interpreted as a first-step lectotypification. In this context, the sheet SGO000004131 is here selected as a second-step lectotype of the name

### 2. Cryptantha alyssoides

Taxon classificationPlantaeBoraginalesBoraginaceae

(A. DC.) Reiche, Anales Univ. Chile 121: 824. 1907.

AFCA916B-B0D2-5156-A13B-918FD4F90F2E

≡ Eritrichiumalyssoides A. DC., Prodr. [A. P. de Candolle] 10: 131. 1846. Krynitzkiaalyssoides (A. DC.) A. Gray, Proc. Am. Acad. 20: 280. 1885. Type: Chile. Región del Libertador General Bernardo O’Higgins: Talcaregué, 1833, *C. Gay s.n.* (holotype: G [G00204936 digital image!]).
= Eritrichiumgilliesii Phil., Anales Univ. Chile 43: 517. 1873. Cryptanthagilliesii (Phil.) Reiche, Fl. Chile 5: 229. 1907. Type: Chile. Región Metropolitana de Santiago: Valle del Yeso, Jan. 1866, *R.A. Philippi s.n.* (first-step lectotype, designated by Johnston 1927, pg. 65: SGO; second-step lectotype, designated here: SGO [SGO000004084 digital image!]; isolectotype: SGO [SGO000004085 digital image!]).
= Eritrichiumtalquinum Phil., Anales Univ. Chile 90: 517. 1895. Cryptanthatalquina (Phil.) Brand, Pflanzenr. (Engler) [Heft 97] 4, Fam. 252: 32. 1931. Type: Chile. Región del Maule: Talca, Feb. 1879, *F. Philippi s.n.* (lectotype, designated by Johnston 1927, pg. 65: SGO [SGO000004140 digital image!]; isolectotypes: CORD [CORD00003778 digital image!], GH [GH00096580 digital image!]).

#### Notes.

In describing *Eritrichiumgilliesii*, Rudolph. A. Philippi (1873) cited a collection he made in Valle del Yeso, Chile. Two specimens of the gathering referred to by Philippi are found at SGO; these duplicates all bear original labels annotated by him with the identification of “E.gilliesii” and agree with the diagnosis cited in the protologue. Johnston (1927) clearly indicated a sheet housed at SGO as the “type”, although more than one specimen is actually lodged there. Thus, Johnston’s choice is here interpreted as a first-step lectotypification. In order to narrow this designation, the sheet SGO000004084 is here selected as a second-step lectotype.

The original material of *Eritrichiumtalquinum*, as referred to by Rudolph A. Philippi (1895) in the protologue of the species, was collected by Friedrich Philippi in Talca, Chile. There are two sheets of apparent original material, which agree with the diagnosis and cited locality, at CORD and SGO. Both specimens were presumably studied by R. A. Philippi since they were annotated, in his hand, as “Eritrichiumtalquinum”. Johnston (1927) noted the specimen at SGO as the “type”, although the author of the name had not indicated any collection as such at the time he published the species. Thus, given that only one element at SGO satisfies the information given by Johnston, his statement is here interpreted as a lectotype designation.

### 3. Cryptantha aprica

Taxon classificationPlantaeBoraginalesBoraginaceae

(Phil.) Reiche, Anales Univ. Chile 121: 814. 1907.

6C4205C4-E0A5-5006-A7C4-C3E72012A4C4

≡ Eritrichiumapricum Phil., Linnaea 33: 190. 1864. Type: Chile. Región de Valparaíso: Catemu, Sep. 1860, *R.A. Philippi s.n.* (lectotype, designated here: SGO [SGO000004035 digital image!]).
= Eritrichiumbridgesii Phil., Anales Univ. Chile 90: 515. 1895. Cryptanthabridgesii (Phil.) Brand, Pflanzenr. (Engler) [Heft 97] 4, Fam. 252: 30. 1931. Type: Chile. Región Metropolitana de Santiago: Lampa, Nov. 1864, *R.A. Philippi s.n.* (first-step lectotype, designated by Johnston 1927. pg. 70: SGO; second-step lectotype, designated here: SGO [SGO000004044 digital image!]; isolectotypes: GH [GH00096364 digital image!], SGO [SGO000004043 digital image!]).

#### Notes.

The protologue of *Eritrichiumapricum* (Philippi 1864) includes a direct reference to various collections made by R. A. Philippi in Aconcagua province and Catemu, Chile. Four syntypes annotated in Philippi’s hand as “Eritrichiumapricum” and in agreement with the diagnosis cited in the protologue are found at GH, S, and SGO. The material held at GH and SGO consists of three specimens collected in Catemu in 1860; one from among them (SGO000004035), however, has a more precise reference to the place of collection (i.e., Cajón del Boldo). On the other hand, the sheet found at S (S12–25271) comes from a collection made in Aconcagua province, Chile, without reference to the date of collection. In this context, the sheet SGO000004035 is here chosen as the lectotype.

In describing *Eritrichiumbridgesii*, Rudolph A. Philippi (1895) cited a collection he made in Lampa, Chile. Three specimens linked to the type collection are found at GH and SGO. The material kept at SGO bears original labels annotated by R. A. Philippi with the identification of “Eritrichiumbridgesii” and agree with the diagnosis as referred to in the protologue. Johnston (1927) clearly indicated by direct citation that the type is housed at SGO with no further reference. In this context, Johnston’s (1927) statement must thus be considered as a first-step typification since two duplicates of the gathering made by R. A. Philippi were found at SGO. In order to narrow this broad designation, the sheet SGO000004044 is here selected as a second-step lectotype (Art. 9.17 of the ICN; Turland et al. 2018).

### 4. Cryptantha argentea

Taxon classificationPlantaeBoraginalesBoraginaceae

I.M. Johnst., Contr. Gray Herb. 78: 42. 1927.

A395E49F-7713-5A62-9E44-BCF2BC272689

#### Type.

Chile. Región de Antofagasta: Antofagasta, about head of high fog-bathed sea-cliffs near Aguada Grande, 16–18 Dec. 1925, *I.M. Johnston 5814* (lectotype, designated here: GH [GH00011520 digital image!]; isolectotypes: E [E00026029 digital image!], GH [GH00011519 digital image!], K [K000573751 digital image!], US [US00111035 digital image!]).

#### Note.

In the protologue of *Cryptanthaargentea*, Johnston (1927) cited a collection he made near Aguada Grande, Chile, and explicitly stated that the type was lodged at GH. Two duplicates of the gathering *Johnston 5814* are found there in agreement with the protologue as referred to by Johnston (1927). Thus, Johnston’s (1927) statement is insufficiently precise since it cannot be ascertained to which of the two specimens at GH he was referring. In this context, the sheet GH00011520 is selected as lectotype of the name.

### 5. Cryptantha capituliflora

Taxon classificationPlantaeBoraginalesBoraginaceae

(Clos) Reiche, Anales Univ. Chile 121: 822. 1907.

9E5632D4-5B69-5634-BBE3-D2DA23E63353

≡ Eritrichiumcapituliflorum Clos, Fl. Chil. 4(4): 467. 1849. Cynoglossospermumcapituliflorum (Clos) Kuntze, Revis. Gen. Pl. 3[3]: 204. 1898. Type: Chile. Región de Coquimbo: “Sur les collines des environs de Los Patos”, s.d., *C. Gay 533* (lectotype, designated here: P [P00606749 digital image!]; isolectotypes: GH [GH00096371 digital image!], P [P00606750 digital image!]).

#### Note.

Clos’ (1849) description of *Eritrichiumcapituliflorum* includes a direct reference to a collection made by Claude Gay in Coquimbo, Chile. Two duplicates of the collection involved are found at P in agreement with the diagnosis and cited locality as referred to by Clos in the protologue. In this context, the duplicate P00606749 is here selected as lectotype of the name.

### 6. Cryptantha chaetocalyx

Taxon classificationPlantaeBoraginalesBoraginaceae

(Phil.) I.M. Johnst., Contr. Gray Herb. 78: 43. 1927.

A3EBB54A-2AFA-5241-9A07-7F3FF6822682

≡ Eritrichiumchaetocalyx Phil., Fl. Atacam. 39. 1860. Type: Chile. Región de Atacama: Caldera, Pan de Azúcar, Dec. 1853, *R.A. Philippi s.n.* (first-step lectotype, designated by Johnston 1927, pg. 43: SGO; second-step lectotype, designated here: SGO [SGO000004073 digital image!]; isolectotypes: GH [GH00096375 digital image!], SGO [SGO000004050 digital image!]).
= Eritrichiumdivaricatum Phil., Anales Univ. Chile 90: 534. 1895. Cryptanthadivaricata (Phil.) Reiche, Anales Univ. Nac. Tec. Altiplano 121: 827. 1907. Type: Chile. Región de Atacama: Caldera, Sep. 1885, *F. Philippi s.n*. (first-step lectotype, designated by Johnston 1927, pg. 43: SGO; second-step lectotype, designated here: SGO [SGO000004070 digital image!]; isolectotypes: GH [GH00096373 digital image!], SGO [SGO000004071 digital image!]).
= Eritrichiumpustulosum Phil., Anales Univ. Chile 90: 537. 1895. Type: Chile. Región de Atacama: Caldera, Sep. 1879, *R.. Philippi s.n*. (first-step lectotype, designated by Johnston 1927, pg. 43: SGO; second-step lectotype, designated here: SGO [SGO000004125 digital image!]; isolectotypes: GH [GH00096374 digital image!], SGO [SGO000004126 digital image!], SGO [SGO000004127 digital image!]).

#### Notes.

In describing *Eritrichiumchaetocalyx*, Rudolph A. Philippi (1860) cited a gathering he made near Pan de Azúcar, Chile. Two duplicates of the type collection in agreement with the protologue and annotated by him as “Eritrichiumchaetocalyx” were located at SGO. Johnston (1927) discussed this name and its original material, referring to a sheet lodged at SGO as the “type” of the name. In this context, Johnston’s (1927) statement must thus be considered as a first-step typification. Thus, in order to narrow this designation, the sheet SGO000004073 is here selected as a second-step lectotype.

In the protologue of *Eritrichiumdivaricatum*, Rudolph A. Philippi (1895) cited as type material a collection made by F. Philippi in Caldera, Chile. Johnston (1927) referred to a specimen kept at SGO as the “type” of the name, although two sheets annotated, in R. A. Philippi’s hand, as “Eritrichiumdivaricatum” are found there. This situation determines that Johnston’s (1927) statement must be considered as a first-step lectotypification. In this context, the duplicate SGO000004070 is here selected as a second-step lectotype (Art. 9.17 of the ICN; Turland et al. 2018).

Rudolph A. Philippi (1895) described *Eritrichiumpustulosum* based on a specimen he collected in Caldera, Chile. According to Johnston (1927), the type material of the name is lodged at SGO. However, three sheets linked to *E.pustulosum*, in agreement with the protologue, were located at SGO, which can certainly be considered as original material. In this context, Johnston’s (1927) statement must be considered as a first-step lectotypification. In order to narrow this earlier designation, the sheet SGO000004125 is here selected as a second-step lectotype.

### 7. Cryptantha cynoglossoides

Taxon classificationPlantaeBoraginalesBoraginaceae

(Phil.) I.M. Johnst., Contr. Gray Herb. 78: 67. 1927.

28224890-3A25-56F1-9F1B-2571CE461646

≡ Eritrichiumcynoglossoides Phil., Linnaea 29: 16. 1858. Type: Chile. Región de Coquimbo: Arqueros, Oct. 1836, *C. Gay s.n*. (holotype: SGO [SGO000004057 digital image!]).
= Eritrichiumuspallatense Phil., Anales Univ. Chile 90: 521. 1895. Type: Argentina. Mendoza: Baños del Inca, Jan. 1886, *A. Borchers s.n*. (first-step lectotype, designated by Johnston 1927, pg. 68: SGO; second-step lectotype, designated here: SGO [SGO000004144 digital image!]; GH [GH00096583 digital image!]; SGO [SGO000004145 digital image!]).

#### Note.

In the protologue of *Eritrichiumuspallatense*, Rudolph A. Philippi (1895) indicated that his diagnosis was based on material collected by Augusto Borchers in Baños del Inca, Mendoza, Argentina. Johnston (1927) extensively revised the type collection linked to this name and stated by direct citation that the type is housed at SGO. However, two duplicates were found there in agreement with the protologue. Consequently, Johnston’s (1927) statement must be considered as a first-step typification. In order to narrow his designation, the specimen SGO000004144 is here selected as a second-step lectotype (Art. 9.17 of the ICN, Turland et al. 2018).

### 8. Cryptantha dichita

Taxon classificationPlantaeBoraginalesBoraginaceae

(Phil.) I.M. Johnst., Contr. Gray Herb. 78: 35. 1927.

F88B7F6F-30B9-5F60-AF8E-E26D125C34E0

≡ Eritrichiumdichita Phil., Anales Univ. Chile 90: 516. 1895. Type: Chile. [Unknown region:] Desierto de Atacama, 1877, *A. Villanueva s.n*. (first-step lectotype, designated by Johnston 1927, pg. 35: SGO; second-step lectotype, designated here: SGO [SGO000004062 image!; isolectotypes: GH [GH00096381 digital image!], SGO [SGO000004063 digital image!]).

#### Note.

In describing *Eritrichiumdichita*, Rudolph A. Philippi (1895) cited eight specimens received from Augusto Villanueva from the Atacama Desert, Chile. However, only two specimens are currently lodged at SGO, where R. A. Philippi worked (Stafleu and Cowan 1983). Both specimens are annotated, in Philippi’s hand, as “Eritrichiumdichita” and in agreement with the diagnosis found in the protologue. Johnston (1927) referred to a sheet housed at SGO as the “type” of the species name, although Philippi did not indicate any material as such at the time he published the species. Thus, Johnston’s (1927) statement must be considered as a first-step lectotypification. In order to narrow this broad designation, the specimen SGO000004062 is here selected as a second-step lectotype.

### 9. Cryptantha diffusa

Taxon classificationPlantaeBoraginalesBoraginaceae

(Phil.) I.M. Johnst., Contr. Gray Herb. 78: 52. 1927.

98C2FE90-A1AE-5167-9332-AA2856FDBE05

Cryptantha diffusa ≡ Eritrichiumdiffusum Phil., Linnaea 33: 191. 1864. Type: Chile. Región de Coquimbo: “Huanta, Baños del Toro”, 1860–1861, *H. Volckmann s.n*. (first-step lectotype, designated by Johnston 1927, pg. 53: SGO; second-step lectotype, designated here, SGO [bc] SGO000004064 image!; isolectotype, SGO [bc] SGO000004065 image!).
= Eritrichiummicranthum Phil., Fl. Atac. 38. 1860. Type: Chile. [Región de Antofagasta:] Sandón, Feb. 1854, *R.A. Philippi s.n*. (first-step lectotype, designated by Johnston 1927, pg. 53: SGO; second-step lectotype, designated here: SGO [SGO000004107 digital image!]; isolectotype: SGO [SGO000004106 digital image!]).
= Cryptanthafamatinae Brand, Repert. Spec. Nov. Regni Veg. 20: 317. 1924. Type: Argentina. La Rioja: La Incrucijada [sic], Sierra Famatina, 1879, *G.H.E.W. Hieronymus & G. Niederlein 466* (lectotype, designated here: CORD [CORD00003765 digital image!]; isolectotype: GH [GH00096383 digital image!]).

#### Notes.

The protologue of *Eritrichiumdiffusum* (Philippi 1864) includes a direct reference to a collection made by Herman Volckmann in Coquimbo, Chile. Johnston (1927) referred to a specimen lodged at SGO as the “type” of the name, while Pérez-Moreau (1976) later stated “Chile. Coquimbo: Huanta, Baños del Toro, 1860–61, Volckmann (tipo de *E.diffusum*, SGO)”. Despite the statements of these authors, in the general collection at SGO two sheets were found in agreement with the protologue. The specimens match the diagnosis as coined by Philippi. In this framework, the earliest statement made by Johnston (1927) is here interpreted as a first-step lectotypification. In order to narrow this broad designation, the sheet SGO000004064 is here selected as a second-step lectotype.

Rudolph A. Philippi (1860) described *Eritrichiummicranthum* based on a collection he made in Sandón, Chile, with no further reference. According to Johnston (1927), the type element of this species is housed at SGO. Johnston’s (1927) statement is here interpreted as a first-step lectotype designation since two sheets in agreement with the protologue and annotated by Philippi as “Eritrichiummicranthum” were found at SGO. The specimens involved match the protologue and, thus, the designation of Johnston (1927) is here narrowed by selecting the sheet SGO000004107 as a second step-lectotype.

In the protologue of *Cryptanthafamatinae*, Brand (1924) cited a collection made by Georg H. E. W. Hieronymus and Gustav Niederlein in the province of La Rioja, Argentina. No original collections could be traced at B, where Brand worked (Stafleu and Cowan 1976), but duplicates of Hieronymus and Niederlein’s collection were located at CORD and GH. These sheets agree with the diagnosis and cited locality found in the protologue. Thus, the sheet CORD00003765 is here selected as lectotype of the name.

### 10. Cryptantha dimorpha

Taxon classificationPlantaeBoraginalesBoraginaceae

(Phil.) Greene, Pittonia 1(3): 112. 1887.

5864A659-7320-5FDB-A728-DEB1385952F2

≡ Eritrichiumdimorphum Phil., Linnaea 29: 16. 1857. Type: Chile. Región Metropolitana de Santiago: Cordillera de Santiago, Feb. 1857, *R.A. Philippi s.n*. (lectotype, designated by Johnston 1927, pg. 67: SGO [SGO000004066 digital image!]; isolectotypes: CORD [CORD00003771 digital image!], GH [GH00096384 digital image!], S [S no. 12–25247 digital image!]).

#### Note.

Rudolph A. Philippi (1857) based the diagnosis of *Eritrichiumdimorphum* on a collection he made in Santiago, Chile, without explicit further reference to any locality. Johnston (1927), in his revision of *Cryptantha* in South America, referred to a specimen lodged at SGO as the “type” of the name. However, an additional duplicate annotated, in Philippi’s hand, as “Eritrichiumdimorphum”, is found at CORD. Both specimens are in agreement with the diagnosis as referred to by Philippi (1857). Therefore, the element cited by Johnston and his statement must be considered as an effective lectotypification of the name.

### 11. Cryptantha filaginea

Taxon classificationPlantaeBoraginalesBoraginaceae

Reiche, Anales Univ. Chile 121: 829. 1907.

CB06C2A9-3F8D-5F4A-ADA6-262DB0B29895

≡ Eritrichiumfilagineum Phil., Anales Univ. Chile 90: 536. 1895. Type: Chile. Región de Atacama: Monte Amargo, Sep. 1885, *F. Philippi s.n.* (first-step lectotype, designated by Johnston 1927, pg. 47: SGO; second-step lectotype, designated here: SGO [SGO000004075 digital image!]; isolectotypes: GH [GH00096386 digital image!], SGO [SGO000004074 digital image!]).

#### Note.

The protologue of *Eritrichiumfilagineum* (Philippi, 1895) includes a direct reference to a collection made by F. Philippi between Caldera and Copiapó, Chile. Two duplicates of a collection in agreement with the protologue and annotated, in R. A. Philippi’s hand, as “Eritrichiumfilagineum” were found at SGO. According to Johnston (1927), the type element of this species is housed at SGO, and therefore his statement is here interpreted as a first-step lectotype designation. In this context, the duplicate SGO000004075 is here selected as a second-step lectotype of the name.

### 12. Cryptantha globulifera

Taxon classificationPlantaeBoraginalesBoraginaceae

(Clos) Reiche, Anales Univ. Chile 121: 827. 1907.

1CE65947-557F-5AFF-AE31-667AF5188800

≡ Eritrichiumglobuliferum Clos, Fl. Chil. 4(4): 464. 1849. Type: Chile. Región de Coquimbo: Coquimbo, 1836, *C. Gay 47* (first-step lectotype, designated by Pérez-Moreau 1976, pg. 175: P; second-step lectotype, designated here: P [P00606762 digital image!]; isolectotypes: GH [GH00096393 digital image!], P [P00606763 digital image!]).
= Eritrichiumglareosum Phil., Linnaea 33: 189. 1864. Cryptanthaglareosa Greene, Pittonia 1(3): 111. 1887. Type: Chile. Región de Valparaíso: “In alveo fluminis Aconcagua prope San Felipe”, Sep. 1860, *R.A. Philippi s.n.* (first-step lectotype, designated by Johnston 1927, pg. 52: SGO; second-step lectotype, designated here: SGO [SGO000004086 digital image!]; isolectotypes: GH [GH00096392 digital image!], SGO [SGO000004087 digital image!]).
= Eritrichiumcarrizalense Phil., Anales Univ. Chile 90: 526. 1895. Cryptanthacarrizalensis (Phil.) Reiche, Fl. Chile 5: 224. 1907. Type: Chile. Región de Atacama: Yerba Buena, 1885, *R. Godoy de Collao s.n*. (lectotype, designated by Johnston 1927, pg. 52: SGO [SGO000004049 digital image!]; isolectotype: GH [GH00096372 digital image!]).
= Eritrichiumsphaerophorum Phil., Anales Univ. Chile 90: 539. 1895. Type: Chile. Región de Atacama: Caldera, Sep. 1879, *R.A. Philippi s.n.* (first-step lectotype, designated by Johnston 1927, pg. 52: SGO; second-step lectotype, designated here: SGO [SGO000004137 digital image!]; isolectotypes: GH [GH00096576 digital image!]; SGO [SGO000004136 digital image!]).

#### Notes.

Clos’ (1849) described *Eritrichiumglobuliferum* based on two syntypes from Chile. The first collection was made by C. Gay (no. *47*) in Coquimbo, while the other one was also made by him (*s.n.*) in Copiapó. Pérez-Moreau (1976) discussed this name and its original material, stating “Chile. Coquimbo, común en dunas de la costa, La Serena, C. Gay 47, IX-1836 (tipo de *E.globuliferum*, P)”. However, Pérez-Moreau’s statement is insufficiently precise since it cannot be ascertained to which of the two specimens at P he was referring. Thus, his choice is here interpreted as a first-step lectotypification. In order to narrow this designation, the specimen P00606762 is here selected as a second-step lectotype.

In describing *Eritrichiumglareosum*, Rudolph A. Philippi (1864) cited a collection made in “alveo fluminis Aconcagua”, Chile. Two specimens of this collection in agreement with the protologue are found at SGO. These duplicates all bear original labels annotated by Philippi with the identification of “E.glareosum” and agree with the diagnosis cited in the protologue. Johnston (1927) referred to a sheet lodged at SGO as the “type” of *E.glareosum* and, thus, his statement is here interpreted as a first-step lectotype designation. In order to narrow this earlier designation, the specimen SGO000004086 is here selected as a second-step lectotype.

Rudolph A. Philippi (1895) described *Eritrichiumcarrizalense* based on a Rosario Godoy de Callao collection made in Yerba Buena, Chile. A duplicate of this material, annotated in Philippi’s hand as “Eritrichiumcarrizalense”, was lodged at B and now destroyed but a photograph from Macbride’s Berlin negatives (neg. 17371) is available at F with a copy at GH. Furthermore, there is a duplicate at herbarium SGO and fragments of the syntype at B lodged at GH. Johnston (1927) referred to the original material of this name, stating that the “type” was lodged at SGO; in the protologue of the species, however, there is no indication of any particular specimen as such. Thus, Johnston’s (1927) statement is here interpreted as a lectotype designation.

The protologue of *Eritrichiumsphaerophorum* (Philippi 1895) includes a direct reference to a collection made by Rudolph A. Philippi near Caldera, Chile, in 1879. Johnston (1927) clearly indicated a sheet housed at SGO as the “type”, although an additional element, also annotated in Philippi’s hand as “Eritrichiumsphaerophorum” was located there in agreement with the protologue. In this context, Johnston’s (1927) choice is here interpreted as a first-step lectotypification. In order to narrow this designation, the specimen SGO000004137 is selected as a second-step lectotype.

### 13. Cryptantha glomerulifera

Taxon classificationPlantaeBoraginalesBoraginaceae

(Phil.) I.M. Johnst., Contr. Gray Herb. 78: 55. 1927.

91F1083C-905A-5E3C-8E5F-1C0C1F7390AD

≡ Eritrichiumglomeruliferum Phil., Anales Univ. Chile 90: 521. 1895. Type: Chile. Región de Coquimbo: Las Mollacas, Cordillera de Illapel, 1888, *F. Philippi s.n.* (first-step lectotype, designated by Johnston 1927, pg. 56: SGO; second-step lectotype, designated here: SGO [SGO000004088 digital image!]; isolectotypes: GH [GH00096394 digital image!], SGO [SGO000004089 digital image!]).

#### Note.

The protologue of *Eritrichiumglomeruliferum* (Philippi 1895) includes a direct reference to a collection made by F. Philippi in Las Mollacas, Chile. Two specimens in agreement with the protologue as referred to by R. A. Philippi are found at SGO. Johnston (1927) referred to a specimen kept at SGO as the “type” of the species name, while Pérez-Moreau (1976) later discussed this name and its original material, stating “Chile. Coquimbo, Las Mollacas, Cordillera de Illapel, s/c, 1–1888 (tipo de *E.glomeruliferum*, SGO)”. However, the statements made by the authors are insufficiently precise since it cannot be ascertained to which of the specimens at SGO they were referring. Thus, the earliest choice of Johnston is here interpreted as a first-step lectotypification. In order to narrow this designation, the specimen SGO000004088 is here selected as a second-step lectotype.

### 14. Cryptantha granulosa

Taxon classificationPlantaeBoraginalesBoraginaceae

(Ruiz & Pav.) I.M. Johnst., Contr. Gray Herb. 68: 54. 1923.

D7680245-723A-5988-ACB7-CC4604AAD163

≡ Myosotisgranulosa Ruiz & Pav., Fl. Peruv. 2: 5. 1799. Type: Peru. Lima: “In Chancay collibus”, s.d., *H. Ruiz & J.A. Pavón s.n.* (lectotype, designated here: MA [MA814804 digital image!]).

#### Note.

According to the protologue of *Myosotisgranulosa*, Ruiz López & Pavón (1799) based the description of this species on two syntypes they had collected during their stay in the Americas. The first collection was made in Lima, Peru, while the other one consists of a collection from Chancay, Peru. Two specimens linked to this name were located at MA in full agreement with the diagnosis and localities cited in the protologue. From among the material available for typification purposes, the specimen MA814804 from Chancay is preferred over the material from Lima (MA814805 ) since it bears an original label annotated, in Pavón’s hand, as “Myosotisgranulosa”. Therefore, it is here selected as the lectotype of the name.

### 15. Cryptantha kingii

Taxon classificationPlantaeBoraginalesBoraginaceae

(Phil.) Reiche, Anales Univ. Chile 121: 815. 1907.

09A3C5D8-4EC9-5738-B8A1-BAD61416EDE6

≡ Eritrichiumkingii Phil., Anales Univ. Chile 90: 516. 1895. Type: Chile. Región de Atacama: “Prope Carrizal Bajo”, s.d., *G. King s.n.* (Type not found).
= Eritrichiumvirens Phil., Anales Univ. Chile 90: 519. 1895. Cryptanthavirens (Phil.) Reiche, Anales Univ. Chile 121: 826. 1907. Type: Chile. Región de Atacama: Bandurrias, 1885, *G. Geisse s.n.* (first-step lectotype, designated by Johnston 1927, pg. 72: SGO; second-step lectotype, designated here: SGO [SGO000004149 digital image!]; isolectotype: SGO [SGO000004150 digital image!]).

#### Note.

Rudolph A. Philippi (1895) based the diagnosis of *Eritrichiumvirens* on a collection made by Guillermo Geisse in Copiapó, on the road to Chañarcillo, Chile. Johnston (1927), in his revision of South American *Cryptantha*, referred to a specimen lodged at SGO as the “type” of the name. However, two duplicates studied by Philippi were located at SGO in agreement with the protologue. Thus, Johnston’s choice is here interpreted as a first-step lectotypification. In order to narrow this designation, the specimen SGO000004149 is here selected as a second-step lectotype.

### 16. Cryptantha longifolia

Taxon classificationPlantaeBoraginalesBoraginaceae

(Phil.) Reiche, Anales Univ. Chile 121: 823. 1907.

C0D53126-1329-55E8-A603-FF6758D0493E

≡ Eritrichiumlongifolium Phil., Anales Univ. Chile 90: 522. 1895. Type: Chile. Región del Biobío: La Polcura, Jan. 1888, *R.A. Philippi s.n.* (first-step lectotype, designated by Johnston 1927, pg. 57: SGO; second-step lectotype, designated here: SGO [SGO000004102 digital image!]; isolectotypes: GH [GH00096556 digital image!], SGO [SGO000004103 digital image!]).

#### Note.

Rudolph A. Philippi (1895) based the diagnosis of *Eritrichiumlongifolium* on a collection he made in La Polcura, Chile. Johnston (1927) referred to a specimen lodged at SGO as the “type” of the name. However, two duplicates studied and annotated as “Eritrichiumlongifolium” by Philippi were located there in agreement with the protologue. Thus, Johnston’s (1927) choice is here interpreted as a first-step lectotypification. In order to narrow this broad designation, the specimen SGO000004102 is here selected as a second-step lectotype.

### 17. Cryptantha patagonica

Taxon classificationPlantaeBoraginalesBoraginaceae

(Speg.) I.M. Johnst., Contr. Gray Herb. 68: 54. 1923.

74C39958-F0BE-50D5-A00B-41C2CEDF0199

≡ Amsinckiapatagonica Speg., Nov. Add. Fl. Patag. 39. 1902. Type: Argentina. [Unknown province:] “Hab. in aridis secus Río S. Cruz”, Feb. 1882, *C. Spegazzini s.n.* (lectotype, designated here: GH [GH00096569 digital image!]).
= Eritrichiumparvulum Phil., Anales Univ. Chile 90: 535. 1895. Cryptanthaparvula (Phil.) Brand, Pflanzenr. (Engler) [Heft 97] 4, Fam. 252: 50. 1931. Type: Chile. Región de Atacama: Caldera, Sep. 1885, *F. Philippi s.n.* (lectotype, designated here: SGO [SGO000004116 digital image!]; isolectotypes: CORD [CORD00003777 digital image!], GH [GH00096568 digital image!], SGO [SGO000004115 digital image!]).

#### Notes.

In describing *Amsinckiapatagonica*, Spegazzini (1902) cited a syntype from Patagonia, Argentina. The gathering consists of a collection he made in Río Santa Cruz, Argentina, in 1882. No specimen corresponding to the material cited in the protologue was located at LP, where Spegazzini worked (Stafleu and Cowan 1986). However, a duplicate of the syntype in agreement with the protologue is kept at GH. This material matches the diagnosis coined by Spegazzini, and, thus, it is here chosen as the lectotype of the name. Rudolph A. Philippi (1895) based the description of *Eritrichiumparvulum* on three collections made by F. Philippi on a trip to the Atacama Region, Chile, in 1885. The first gathering consists of a collection from Caldera, while the other two were made in Piedra Colgada and Chañarcillo. Four duplicates of the syntype collected in Caldera are kept at CORD, GH, and SGO. Among the other syntypes, a duplicate of each collection is housed at SGO. All specimens are in agreement with the localities and the diagnosis cited in the protologue. The syntype from Caldera is preferred as it shows the best quality of preservation of the important diagnostic features of the taxon. Thus, a duplicate from this collection is here chosen as lectotype of the name.

### 18. Cryptantha spathulata

Taxon classificationPlantaeBoraginalesBoraginaceae

(Phil.) Reiche, Anales Univ. Chile 121: 823. 1907.

AE0B347C-6D43-551E-A694-D79E71F0CC66

≡ Eritrichiumspathulatum Phil., Anales Univ. Chile 90: 517. 1895. Type: Chile. [Región del Libertador General Bernardo O’Higgins:] Colchagua de Popeta, 1881, *F. Philippi s.n.* (first-step lectotype, designated by Johnston 1927, pg. 58: SGO; second-step lectotype, designated here: SGO [SGO000004135 digital image!]; isolectotypes: GH [GH00096577 digital image!], SGO [SGO000004134 digital image!]).

#### Note.

In describing *Eritrichiumspathulatum*, Rudolph A. Philippi (1895) cited a collection made by F. Philippi in Colchagua de Popeta, Chile. Two duplicates of this gathering in agreement with the protologue and annotated as “Eritrichiumspathulatum” by Philippi are found at SGO. Johnston (1927) referred to the “type” as lodged at SGO and, therefore, his statement must be considered as a first-step lectotypification. In order to narrow this broad designation, the specimen SGO000004135 is here selected as a second-step lectotype.

### 19. Cryptantha subamplexicaulis

Taxon classificationPlantaeBoraginalesBoraginaceae

(Phil.) Reiche, Anales Univ. Chile 121: 826. 1907.

AD866517-023E-5CAC-8DD8-B6BC415265B6

≡ Eritrichiumsubamplexicaule Phil., Fl. Atacam. 39. 1860. Type: Chile. Región de Antofagasta: Paposo, Dec. 1853, *R.A. Philippi s.n.* (lectotype, designated by Johnston 1927, pg. 41: SGO [SGO000004139 digital image!]; isolectotypes: GH [GH00096579 digital image!], HAL [HAL0115278 digital image!]).

#### Note.

In the protologue of *Eritrichiumsubamplexicule*, Rudolph A. Philippi (1860) cited a collection he made near Paposo, Chile. There are two sheets of apparent original material, which agree with the diagnosis and cited locality, at HAL and SGO. Both specimens were presumably studied by Philippi since they were annotated, in his hand, as “Eritrichiumsubamplexicaule”. The material at SGO was referred to as the “type” of the species name by Johnston (1927). Therefore, the element cited by him must be considered as an effective lectotypification.

### 20. Cryptantha volckmannii

Taxon classificationPlantaeBoraginalesBoraginaceae

(Phil.) I.M. Johnst., Contr. Gray Herb. 78: 66. 1927.

4B1BFE67-D60B-5F04-91B8-409D1E3953CC

≡ Eritrichiumvolckmannii Phil., Anales Univ. Chile 18: 54. 1861. Type: Chile. Región de Coquimbo: Huanta, 1860, *H. Volckmann s.n.* (holotype: SGO [SGO000004151 digital image!]).
= Eritrichiumchrysanthum Phil., Linnaea 33: 191. 1864. Cryptanthachrysantha (Phil.) Reiche, Anales Univ. Chile 121: 815. 1907. Type: Chile. Región de Coquimbo: Cordillera de Illapel, Aug. 1861, *H. Volckmann s.n.* (first-step lectotype, designated by Johnston 1927, pg. 66: SGO; second-step lectotype, designated here: SGO [SGO000004051 digital image!]; isolectotypes: GH [GH00096376 digital image!], SGO [SGO000004052 digital image!]).

#### Note.

Rudolph A. Philippi’s description of *Eritrichiumchrysanthum* (Philippi 1864) was based on material collected by H. Volckmann near Illapel, Chile. Johnston (1927) indicated a specimen housed at SGO as the “type”. However, two sheets linked to *E.chrysanthum* were located at SGO and, therefore, his statement must be considered as a first-step lectotypification. In order to narrow this broad designation, the specimen SGO000004051 is here selected as a second-step lectotype.

## Supplementary Material

XML Treatment for Cryptantha alfalfalis

XML Treatment for Cryptantha alyssoides

XML Treatment for Cryptantha aprica

XML Treatment for Cryptantha argentea

XML Treatment for Cryptantha capituliflora

XML Treatment for Cryptantha chaetocalyx

XML Treatment for Cryptantha cynoglossoides

XML Treatment for Cryptantha dichita

XML Treatment for Cryptantha diffusa

XML Treatment for Cryptantha dimorpha

XML Treatment for Cryptantha filaginea

XML Treatment for Cryptantha globulifera

XML Treatment for Cryptantha glomerulifera

XML Treatment for Cryptantha granulosa

XML Treatment for Cryptantha kingii

XML Treatment for Cryptantha longifolia

XML Treatment for Cryptantha patagonica

XML Treatment for Cryptantha spathulata

XML Treatment for Cryptantha subamplexicaulis

XML Treatment for Cryptantha volckmannii
